# Developing a prediction model for all‐cause mortality risk among patients with type 2 diabetes mellitus in Shanghai, China

**DOI:** 10.1111/1753-0407.13343

**Published:** 2022-12-16

**Authors:** Jiying Qi, Ping He, Huayan Yao, Yanbin Xue, Wen Sun, Ping Lu, Xiaohui Qi, Zizheng Zhang, Renjie Jing, Bin Cui, Guang Ning

**Affiliations:** ^1^ Department of Endocrine and Metabolic Diseases Shanghai Institute of Endocrine and Metabolic Diseases, Ruijin Hospital, Shanghai Jiao Tong University School of Medicine Shanghai China; ^2^ Shanghai National Clinical Research Center for Metabolic Diseases, Key Laboratory for Endocrine and Metabolic Diseases of the National Health Commission of the PR China, Shanghai Key Laboratory for Endocrine Tumor, State Key Laboratory of Medical Genomics Ruijin Hospital, Shanghai Jiao Tong University School of Medicine Shanghai China; ^3^ Link Healthcare Engineering and Information Department, Shanghai Hospital Development Center Shanghai China; ^4^ Computer Net Center, Ruijin Hospital, Shanghai Jiao Tong University School of Medicine Shanghai China; ^5^ Wonders Information Co. Ltd. Shanghai China

**Keywords:** all‐cause mortality, prediction model, type 2 diabetes mellitus, 全因死亡率, 预测模型, 2型糖尿病

## Abstract

**Background:**

All‐cause mortality risk prediction models for patients with type 2 diabetes mellitus (T2DM) in mainland China have not been established. This study aimed to fill this gap.

**Methods:**

Based on the Shanghai Link Healthcare Database, patients diagnosed with T2DM and aged 40‐99 years were identified between January 1, 2013 and December 31, 2016 and followed until December 31, 2021. All the patients were randomly allocated into training and validation sets at a 2:1 ratio. Cox proportional hazards models were used to develop the all‐cause mortality risk prediction model. The model performance was evaluated by discrimination (Harrell C‐index) and calibration (calibration plots).

**Results:**

A total of 399 784 patients with T2DM were eventually enrolled, with 68 318 deaths over a median follow‐up of 6.93 years. The final prediction model included age, sex, heart failure, cerebrovascular disease, moderate or severe kidney disease, moderate or severe liver disease, cancer, insulin use, glycosylated hemoglobin, and high‐density lipoprotein cholesterol. The model showed good discrimination and calibration in the validation sets: the mean C‐index value was 0.8113 (range 0.8110–0.8115) and the predicted risks closely matched the observed risks in the calibration plots.

**Conclusions:**

This study constructed the first 5‐year all‐cause mortality risk prediction model for patients with T2DM in south China, with good predictive performance.

## Graphical abstract

**Highlights**

- This study constructed the first 5‐year all‐cause mortality risk prediction model for patients with type 2 diabetes mellitus in south China.
- This well‐performing model, using highly accessible predictors collected in routine clinical settings, provides a powerful tool for clinicians to identify high‐risk diabetic patients.

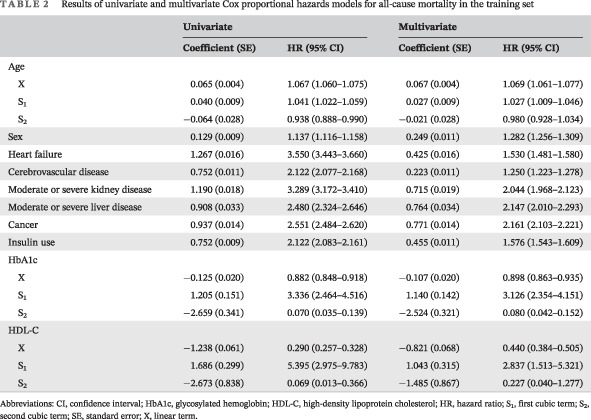

## 摘要

**背景:** 中国大陆地区2型糖尿病(T2DM)患者全因死亡风险预测模型尚未建立, 本项研究旨在填补这一空白。

**方法:** 基于上海申康医疗数据库, 筛选出2013年1月1日至2016年12月31日期间年龄在40‐99岁的T2DM患者, 随访至2021年12月31日。所有患者按2:1的比例随机分配到训练组和验证组。采用Cox比例风险模型建立全因死亡风险预测模型。通过鉴别(Harrell c指数)和校准(校准图)对模型性能进行评价。

**结果:** 在中位随访6.93年期间, 共有399784例T2DM患者最终入选, 其中68318例死亡。最终的预测模型包括年龄、性别、心力衰竭、脑血管疾病、中度或重度肾病、中度或重度肝病、癌症、胰岛素使用、HbA_1c_和高密度脂蛋白‐胆固醇。模型在验证组中表现出良好的判别能力, 平均c指数值为0.8113(范围为0.8110‐0.8115)。在校准图中, 预测的风险与观测到的风险密切匹配, 表明模型校准良好。

**结论:** 本研究构建了中国南方地区首个T2DM患者5年全因死亡风险预测模型, 具有较好的预测性能。

## 1 INTRODUCTION

Diabetes is a chronic metabolic disease that severely threatens human health. More than 6.7 million adults were estimated to have died from diabetes or its complications in 2021, accounting for 12.2% of all‐cause deaths worldwide.^1^ Previous studies have noted that the all‐cause mortality in patients with diabetes is almost twice that of those without diabetes; furthermore, the younger the age and the longer the duration of diabetes, the higher the risk of mortality.^2^, ^3^, ^4^, ^5^, ^6^, ^7^ Early screening of high‐risk diabetic patients is undoubtedly crucial so that we can enhance clinical management and provide timely intervention, thereby reducing the risk of premature mortality. One of the practical and effective approaches is to develop all‐cause mortality risk prediction models for the diabetic population.

In the past decade or so, many prediction models have been well developed in Western populations, such as the Estimation of Mortality Risk in Type 2 Diabetic Patients (ENFORCE) model,^8^, ^9^ the Risk Equations for Complications of Type 2 Diabetes (RECODe) model,^10^ the Building, Relating, Assessing, and Validating Outcomes (BRAVO) model,^11^ and the UK Prospective Diabetes Study (UKPDS) Outcomes Model 2 model.^12^ Due to differences in genetics, socioeconomic factors, and diabetes management approaches, these prediction models developed based on Western populations are often not directly applicable to Asian populations. Compared with other populations, Asian populations develop diabetes at a younger age, have a higher risk of complications, suffer longer from complications, and die earlier.^7^, ^13^, ^14^, ^15^ In contrast, prediction models constructed based on Asian populations are still limited, all from Hong Kong^16^, ^17^, ^18^, ^19^ and Taiwan^20^, ^21^, ^22^ in China. To date, there are no relevant studies in mainland China. Therefore, we conducted this study to develop a 5‐year all‐cause mortality prediction model based on a large‐scale population with type 2 diabetes mellitus (T2DM) in Shanghai, China.

## 2 METHODS

### 2.1 Data source

This study was conducted using the Shanghai Link Healthcare Database (SLHD), a representative clinical database covering ＞99% of the residents, developed and operated by the Shanghai Hospital Development Center (an administrative department of the Shanghai Municipal People's Government). In China, government‐run hospitals are classified as primary (grade I), secondary (grade II), and tertiary (grade III) hospitals according to their capabilities in medical care, medical education, and medical research, with tertiary hospitals being the best. The Shanghai Hospital Development Center is responsible for monitoring 35 tertiary hospitals, all of which are required by administrative regulations to upload general medical practice data (i.e., outpatient visits, emergency department visits, and hospital admissions) to the SLHD. The SLHD has released data for academic research since 2013, which requires review and approval to access. Mortality data were obtained from the Shanghai Big Data Center.

Any personally identifiable information was scrambled to protect privacy, so the study was exempt from institutional review board approval because the researchers were blinded to patient identities. All diseases were identified according to the *International Classification of Diseases, 10th Revision* and relevant diagnosis (Table S1).

### 2.2 Study population

First, we identified patients aged 40–99 years who were diagnosed with T2DM between January 1, 2013 and December 31, 2016 (*n* = 418 730). Follow‐up started from the date of the first diagnosis of T2DM until death or December 31, 2021, whichever came first. After excluding patients with <1 year of follow‐up (*n* = 18 946), a total of 399 784 patients with T2DM were finally enrolled in the study (Figure S1).

### 2.3 Candidate predictors

Candidate predictors were selected based on data availability and clinical relevance, including age, sex, hypertension, dyslipidemia, diabetic complications, ischemic heart disease, peripheral vascular disease, heart failure, cerebrovascular disease, dementia, chronic lung disease, moderate or severe kidney disease, mild liver disease, moderate or severe liver disease, cancer, insulin, oral antidiabetic drugs, antihypertensive drugs, lipid‐lowering drugs, anticoagulant drugs, aspirin, other antiplatelet drugs, nonsteroidal anti‐inflammatory drugs, glycosylated hemoglobin (HbA1c), total cholesterol, high‐density lipoprotein cholesterol (HDL‐C), low‐density lipoprotein cholesterol (LDL‐C), and triglyceride. Comorbidities and medications were assessed between the earliest date of recording and the end date of the 1‐year lag period after cohort entry. Biochemical indicators were defined as the closest recorded values within 2 years before and after enrollment.

Multiple imputation was performed to handle missing values of biochemical indicators. The imputation model included candidate predictors and event indicators. A total of 10 datasets were imputed, and the data distribution of biochemical indicators in the original and imputed datasets indicated good imputation (Table S2). Subsequent analyses were repeated for each imputed dataset. Based on asymptotic theory, Rubin developed a set of rules to combine the estimates and standard errors (SEs) of each imputed dataset into an overall estimate and SE to provide valid statistical results.^23^, ^24^ The results of this study were combined by Rubin's rule or shown as the median, interquartile range (IQR), or full range of the 10 estimates.^23^, ^24^

### 2.4 Statistical analysis

All the patients were randomly allocated into training and validation sets at a 2:1 ratio. Baseline characteristics between the training and validation sets were compared using the Student's *t* test for continuous variables and the chi‐square test for categorical variables. Standardized mean differences (SMDs) were also calculated to assess the comparability of baseline characteristics between the training and validation sets, with values less than 10% indicating relative balance.

Cox proportional hazards models were used to develop the all‐cause mortality prediction model. To account for the nonlinearity of the continuous variables (age, HbA1c, total cholesterol, HDL‐C, and LDL‐C), we transformed them using restricted cubic splines with four knots placed at the respective 5th, 35th, 65th, and 95th sample percentiles.^25^ Variable selection was performed within each imputed training dataset. Initially, univariate Cox proportional hazards models were used to identify significant predictors. The major predictors were retained directly to form the basic model, including age and sex. Starting from the basic model, an additional candidate predictor was selected for inclusion in the multivariate Cox proportional hazards model at each step, and then the model was evaluated for improvement. Predictors incorporated into the model should significantly improve discrimination and integrated discrimination improvement, and reduce the Akaike information criterion. The Akaike information criterion is a measure of the model's goodness of fit, with a lower value indicating a better fit.^26^ If a predictor was retained in at least 8/10 of the imputed training datasets, it would be eventually included. Interaction terms between age and other factors and between sex and other factors were also assessed, which did not significantly improve model performance. Predictors included in the final model were age, sex, heart failure, cerebrovascular disease, moderate or severe kidney disease, moderate or severe liver disease, cancer, insulin use, HbA1c, and HDL‐C. Based on the selected variables, models were fitted in each of the 10 imputed training datasets. The estimates were combined using Rubin's rule to obtain coefficients and SEs, as well as hazard ratios (HRs) and 95% confidence intervals (CIs).

The developed model was applied to the 10 imputed validation datasets to evaluate model performance. Discrimination was assessed by estimating Harrell C‐index, with higher values indicating better performance. Calibration was assessed by plotting the predicted event probabilities against the observed event probabilities. The risk prediction model was internally validated using 100 bootstrap samples. In addition, to examine the effect of missing data, we repeated the validation using complete case analysis. All statistical analyses were performed using R language software, version 4.1.2 (R Foundation for Statistical Computing, Vienna, Austria).

## 3 RESULTS

A total of 399 784 patients with T2DM were enrolled in this study. During a median follow‐up of 6.93 years (IQR 5.64–8.31 years), 68 318 patients died, with an all‐cause mortality of 2.54 per 100 person‐years. The median age was 63 years (IQR 56–72 years), and 51.11% were male. Table 1 shows the baseline characteristics of patients with T2DM in the training set (*n* = 266 523) and validation set (*n* = 133 261). The SMD values for all baseline characteristics between the training and validation sets were much lower than 10%, implying that the two were well balanced.

**TABLE 1:** Baseline characteristics of the study population

Variables	Training set (*n* = 266 523)	Validation set (*n* = 133 261)	*p* value	SMD
Age, median (IQR)	63 (56–72)	63 (56–72)	0.981	0.001
Male, *n* (%)	136 408 (51.18)	67 905 (50.96)	0.182	0.004
Comorbidities, *n* (%)				
Hypertension	112 973 (42.39)	56 736 (42.58)	0.260	0.004
Dyslipidemia	33 767 (12.67)	16 873 (12.66)	0.948	<0.001
Diabetic complications	34 522 (12.95)	17 526 (13.15)	0.079	0.006
Ischemic heart disease	51 025 (19.14)	25 598 (19.21)	0.630	0.002
Peripheral vascular disease	4333 (1.63)	2163 (1.62)	0.961	<0.001
Heart failure	10 268 (3.85)	5033 (3.78)	0.243	0.004
Cerebrovascular disease	40 491 (15.19)	20 418 (15.32)	0.285	0.004
Dementia	2244 (0.84)	1096 (0.82)	0.535	0.002
Chronic lung disease	24 764 (9.29)	12 482 (9.37)	0.445	0.003
Moderate or severe kidney disease	7018 (2.63)	3497 (2.62)	0.875	0.001
Mild liver disease	22 718 (8.52)	11 334 (8.51)	0.846	0.001
Moderate or severe liver disease	2698 (1.01)	1375 (1.03)	0.574	0.002
Cancer	19 191 (7.20)	9538 (7.16)	0.623	0.002
Medications, *n* (%)				
Insulin	97 011 (36.40)	48 323 (36.26)	0.399	0.003
Oral antidiabetic drugs	180 598 (67.76)	90 145 (67.65)	0.464	0.002
Antihypertensive drugs	145 440 (54.57)	72 664 (54.53)	0.805	0.001
Lipid‐lowering drugs	94 366 (35.41)	47 086 (35.33)	0.653	0.002
Anticoagulant drugs	5826 (2.19)	2932 (2.20)	0.780	0.001
Aspirin	76 110 (28.56)	38 112 (28.60)	0.780	0.001
Other antiplatelet drugs	46 286 (17.37)	22 946 (17.22)	0.246	0.004
Nonsteroidal anti‐inflammatory drugs	73 898 (27.73)	37 057 (27.81)	0.592	0.002
Biochemical indicators, median (IQR)^a^				
HbA1c (%)	7.100 (6.300–8.400)	7.100 (6.300–8.400)		
Total cholesterol (mmol/L)	4.660 (3.890–5.460)	4.660 (3.890–5.460)		
HDL‐C (mmol/L)	1.140 (0.950–1.380)	1.140 (0.950–1.380)		
LDL‐C (mmol/L)	2.800 (2.170–3.460)	2.800 (2.175–3.460)		
Triglyceride (mmol/L)	1.420 (1.010–2.055)	1.430 (1.010–2.050)		

Abbreviations: HbA1c, glycosylated hemoglobin; HDL‐C, high‐density lipoprotein cholesterol; IQR, interquartile range; LDL‐C, low‐density lipoprotein cholesterol; SMD, standardized mean difference.

^a^ Median of the values of the 10 imputed datasets.

Variable selection was performed in each of the 10 imputed training datasets. Predictors included in the final model were age, sex, heart failure, cerebrovascular disease, moderate or severe kidney disease, moderate or severe liver disease, cancer, insulin use, HbA1c, and HDL‐C. Table 2 shows the results of univariate and multivariate Cox proportional hazards models for all‐cause mortality in the training set, including coefficients (SEs) and HRs (95% CIs). In the multivariate Cox proportional hazards model, all variables were significant except for the second cubic term of the spline function for age and HDL‐C. The 5‐year all‐cause mortality risk prediction equation for patients with T2DM was as follows, where ${\left(u\right)}_{+}=\left\{\begin{array}{c} u,\text{if}\;u>0,\\ 0,\text{if}\;u\leq 0. \end{array}\right.$

$$\begin{array}{l} 100\times (1-0.9980421^{\exp}(0.06667041\times \mathrm{age}+1.860926e^{\left(-05\right)}\\ \;\times {\left(\mathrm{age}-46\right)}_{+}^{3}-1.435202e^{\left(-05\right)}\times {\left(\mathrm{age}-59\right)}_{+}^{3}\\ \;-2.177196e^{\left(-05\right)}\times {\left(\mathrm{age}-68\right)}_{+}^{3}+1.751472e^{\left(-05\right)}\\ \;\times {\left(\mathrm{age}-84\right)}_{+}^{3}+0.24859731\times \left(\text{male}==\text{TRUE}\right)\\ \;+0.42522929\times \left(\text{heart failure}==\text{TRUE}\right)\\ \;+0.22311493\times \left(\text{cerebrovascular disease}==\text{TRUE}\right)\\ \;+0.71501785\times \left(\text{moderate or severe kidney disease}==\text{TRUE}\right)\\ \;+0.76391954\times \left(\text{moderate or severe liver disease}==\text{TRUE}\right)\\ \;+0.77068201\times \left(\text{cancer}==\text{TRUE}\right)\\ \;+0.45461855\times \left(\text{insulin}\;\mathrm{use}==\text{TRUE}\right)\\ \;-0.10718135\times \mathrm{HbA}1c+0.03508194\times {\left(\mathrm{HbA}1c-5.50\right)}_{+}^{3}\\ \;-0.0776974\times {\left(\mathrm{HbA}1c-6.60\right)}_{+}^{3}+0.04498314\\ \;\times {\left(\mathrm{HbA}1c-7.70\right)}_{+}^{3}-0.002367679\times {\left(\mathrm{HbA}1c-11.20\right)}_{+}^{3}\\ \;-0.82047966\times \mathrm{HDL}­C+0.8167382\times {\left(\mathrm{HDL}­C-0.72\right)}_{+}^{3}\\ \;-1.162871\times {\left(\mathrm{HDL}­C-1.02\right)}_{+}^{3}+0.07287736\\ \;\times {\left(\mathrm{HDL}­C-1.27\right)}_{+}^{3}+0.2732556\times {\left(\mathrm{HDL}­C-1.85\right)}_{+}^{3})) \end{array}$$

**TABLE 2:** Results of univariate and multivariate Cox proportional hazards models for all‐cause mortality in the training set

	Univariate		Multivariate	
	Coefficient (SE)	HR (95% CI)	Coefficient (SE)	HR (95% CI)
Age				
X	0.065 (0.004)	1.067 (1.060–1.075)	0.067 (0.004)	1.069 (1.061–1.077)
S_1_	0.040 (0.009)	1.041 (1.022–1.059)	0.027 (0.009)	1.027 (1.009–1.046)
S_2_	−0.064 (0.028)	0.938 (0.888–0.990)	−0.021 (0.028)	0.980 (0.928–1.034)
Sex	0.129 (0.009)	1.137 (1.116–1.158)	0.249 (0.011)	1.282 (1.256–1.309)
Heart failure	1.267 (0.016)	3.550 (3.443–3.660)	0.425 (0.016)	1.530 (1.481–1.580)
Cerebrovascular disease	0.752 (0.011)	2.122 (2.077–2.168)	0.223 (0.011)	1.250 (1.223–1.278)
Moderate or severe kidney disease	1.190 (0.018)	3.289 (3.172–3.410)	0.715 (0.019)	2.044 (1.968–2.123)
Moderate or severe liver disease	0.908 (0.033)	2.480 (2.324–2.646)	0.764 (0.034)	2.147 (2.010–2.293)
Cancer	0.937 (0.014)	2.551 (2.484–2.620)	0.771 (0.014)	2.161 (2.103–2.221)
Insulin use	0.752 (0.009)	2.122 (2.083–2.161)	0.455 (0.011)	1.576 (1.543–1.609)
HbA1c				
X	−0.125 (0.020)	0.882 (0.848–0.918)	−0.107 (0.020)	0.898 (0.863–0.935)
S_1_	1.205 (0.151)	3.336 (2.464–4.516)	1.140 (0.142)	3.126 (2.354–4.151)
S_2_	−2.659 (0.341)	0.070 (0.035–0.139)	−2.524 (0.321)	0.080 (0.042–0.152)
HDL‐C				
X	−1.238 (0.061)	0.290 (0.257–0.328)	−0.821 (0.068)	0.440 (0.384–0.505)
S_1_	1.686 (0.299)	5.395 (2.975–9.783)	1.043 (0.315)	2.837 (1.513–5.321)
S_2_	−2.673 (0.838)	0.069 (0.013–0.366)	−1.485 (0.867)	0.227 (0.040–1.277)

Abbreviations: CI, confidence interval; HbA1c, glycosylated hemoglobin; HDL‐C, high‐density lipoprotein cholesterol; HR, hazard ratio; S_1_, first cubic term; S_2_, second cubic term; SE, standard error; X, linear term.

Example: 65 years old; male; history of heart failure, cerebrovascular disease, moderate or severe kidney disease, moderate or severe liver disease, and cancer; use of insulin; HbA1c level = 12.00%, HDL‐C level = 1.00 mmol/L.

5‐year all‐cause mortality risk ≈ 86.94%

$$\begin{array}{l} 100\times (1-0.9980421^{\exp}(0.06667041\times 65+1.860926e^{\left(-05\right)}\\ \;\times {\left(65-46\right)}_{+}^{3}-1.435202e^{\left(-05\right)}\times {\left(65-59\right)}_{+}^{3}\\ \;+0.24859731+0.42522929+0.22311493\\ \;+0.71501785+0.76391954+0.77068201+0.45461855\\ \;-0.10718135\times 12.00+0.03508194\times {\left(12.00-5.50\right)}_{+}^{3}\\ \;-0.0776974\times {\left(12.00-6.60\right)}_{+}^{3}+0.04498314\\ \;\times {\left(12.00-7.70\right)}_{+}^{3}-0.002367679\times {\left(12.00-11.20\right)}_{+}^{3}\\ \;-0.82047966\times 1.00+0.8167382\times {\left(1.00-0.72\right)}_{+}^{3})) \end{array}$$

**FIGURE 1:**
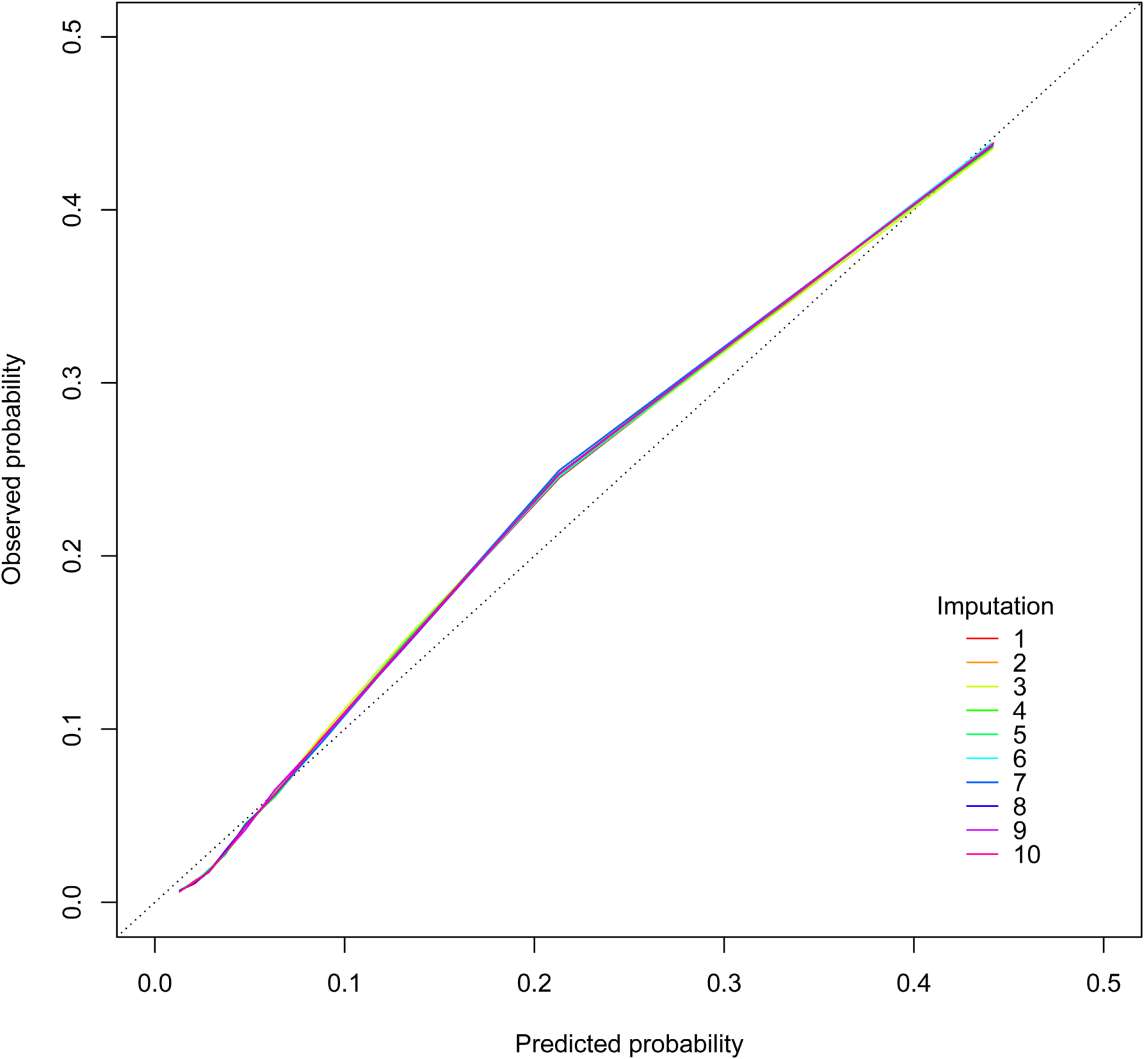
Calibration curves of the 5‐year all‐cause mortality risk prediction model in the imputed validation datasets

Table 3 shows the C‐index of the 10 imputed validation datasets and complete dataset. The prediction model had good discrimination in all imputed validation datasets, with a mean C‐index value of 0.8113 (range 0.8110–0.8115). In the internal validation of 100 bootstrap samples, the mean C‐index value was 0.8113 (range 0.8110–0.8116). Figure 1 shows the calibration curves of the 5‐year all‐cause mortality risk prediction model in the 10 imputed validation datasets. All calibration curves were very close to each other. Although the risk in the middle part was slightly underestimated, overall, there was no significant difference between the predicted and observed risks, indicating that the prediction model was well calibrated. Sensitivity analysis using complete case analysis included 155 354 patients with T2DM, which also observed good discrimination (C‐index 0.8087) and calibration (Figure S2).

**TABLE 3:** C‐index of the imputed validation datasets and complete dataset

	C‐index	95% CI	100 bootstrap C‐index
Imputed validation datasets			
1	0.8114	(0.8088–0.8140)	0.8114
2	0.8113	(0.8087–0.8140)	0.8114
3	0.8113	(0.8087–0.8140)	0.8113
4	0.8112	(0.8085–0.8138)	0.8112
5	0.8110	(0.8083–0.8136)	0.8110
6	0.8111	(0.8085–0.8138)	0.8111
7	0.8114	(0.8087–0.8140)	0.8114
8	0.8113	(0.8086–0.8139)	0.8113
9	0.8115	(0.8088–0.8141)	0.8115
10	0.8115	(0.8089–0.8142)	0.8116
Completed dataset	0.8087	(0.8063–0.8112)	0.8088

Abbreviation: CI, confidence interval.

## 4 DISCUSSION

This study constructed the first 5‐year all‐cause mortality risk prediction model for patients with T2DM in south China. The model achieved good discrimination and calibration by using highly accessible predictors collected in routine clinical settings, including age, sex, heart failure, cerebrovascular disease, moderate or severe kidney disease, moderate or severe liver disease, cancer, insulin use, HbA1c, and HDL‐C.

Over the past decade or so, many all‐cause mortality risk prediction models have been constructed for patients with T2DM. However, due to differences in ethnicity, genetics, socioeconomic factors, and disease management approaches, prediction models developed based on specific populations are usually not directly applicable to other populations. Several models have been established to predict the risk of all‐cause mortality in Western populations with T2DM.^8^, ^9^, ^10^, ^11^, ^12^, ^27^, ^28^, ^29^, ^30^, ^31^ Although some of these models were developed based on multiethnic populations,^10^, ^11^, ^12^, ^28^, ^29^, ^30^, ^31^ the vast majority included ethnicity as a significant predictor.^10^, ^12^, ^28^, ^29^, ^30^, ^31^ Previous studies have pointed out some unique characteristics of Asian diabetic populations, such as younger age of onset of diabetes, higher risk of complications, and earlier time of death.^13^, ^14^, ^15^ Nevertheless, the prediction models developed based on Asian populations cover very few regions, only Hong Kong and Taiwan in China.^16^, ^17^, ^18^, ^19^, ^20^, ^21^, ^22^ It is necessary to construct specific all‐cause mortality risk prediction models for patients with T2DM in mainland China, which could provide more accurate risk prediction.

Notably, the data sources used to develop the models can also affect predictive validity. Whether models developed based on non‐real‐world data can be well applied to the real world remains a question. It has been found that prediction models perform much less well in the clinical trial setting than in the real world.^8^, ^9^, ^10^, ^32^ This may be related to differences in patients' intrinsic motivation and health awareness. Volunteers participating in clinical trials may place greater value on health, which to some extent reduces their mortality risk, thus affecting model performance. In this study, we developed the risk prediction model based on real‐world data to ensure its effectiveness when applied in real‐world clinical settings.

The SLHD is the largest medical database in mainland China, which provided a good basis for the design and implementation of this study. We identified a number of candidate predictors based on data availability and clinical relevance. The final prediction model included age, sex, heart failure, cerebrovascular disease, moderate or severe kidney disease, moderate or severe liver disease, cancer, insulin use, HbA1c, and HDL‐C, which balanced the predictive validity and parsimony of the model well.

Glycemic control is definitely crucial for patients with T2DM. Clinically, the main indicator used to evaluate glycemic control status is HbA1c, which reflects the average plasma glucose level over the past 2–3 months. Insulin, as the most effective class of antidiabetic drugs, is commonly used in patients whose plasma glucose levels cannot be well controlled by oral antidiabetic drugs. In general, patients with higher HbA1c levels or those using insulin have a longer duration and more severe disease. Therefore, HbA1c and insulin play a crucial role in predicting the risk of all‐cause mortality among patients with T2DM.^8^, ^9^, ^10^, ^11^, ^17^, ^18^, ^19^, ^20^, ^21^, ^22^, ^27^, ^33^, ^34^ In addition, heart failure, cerebrovascular disease, moderate or severe kidney disease, moderate or severe liver disease, and cancer are all major diseases that severely threaten human health and life, and their effects on mortality are well recognized.^10^, ^11^, ^18^, ^19^, ^22^ HDL‐C level is also a common predictor in all‐cause mortality prediction models, which may be related to the fact that it is an important factor for cardiovascular disease.^8^, ^9^, ^10^, ^18^, ^19^ Regular screening, early identification, and active intervention for these diseases should be advocated in order to delay disease progression and reduce the risk of premature death.

This study has some limitations. The first is the data limitation. Although the SLHD is the largest medical database in mainland China, there is a relatively high proportion of missing data for some variables, such as biochemical indicators, duration of diabetes, body mass index, smoking, and alcohol consumption. It is worth noting that such missing data were usually not random and may indicate patient characteristics, health awareness level, and health status. Directly removing observations with missing values would introduce severe bias. Therefore, we performed multiple imputation to handle missing data (widely considered the best approach) and conducted complete case analysis to examine the effect of missing data. Second, due to resource constraints, the prediction model was only internally validated, and further external validation in other populations is advisable. Third, only a 5‐year all‐cause mortality risk prediction model was constructed in this study, and future studies with longer follow‐up periods are needed to update the model to predict the mortality risk at 10, 15, and more years.

In conclusion, this study constructed the first 5‐year all‐cause mortality risk prediction model for patients with T2DM in south China, which achieved good discrimination and calibration by using highly accessible predictors collected in routine clinical settings. The model provides a powerful tool for clinicians to identify high‐risk diabetic patients, which can enhance clinical management and facilitate timely intervention, ultimately improving survival quality and extending life expectancy.

## DISCLOSURE STATEMENT

The authors declare that there is no conflict of interest.

## Supporting information


**Figure S1.** Flowchart showing the selection process of the study population

**Figure S2.** Calibration curve of the 5‐year all‐cause mortality risk prediction model in the complete dataset

**Table S1.** ICD‐10 codes for diseases
**Table S2.** Data distribution of biochemical indicators in the original and imputed datasets
