# Supplementary figures and images for: Developing a prediction model for all‐cause mortality risk among patients with type 2 diabetes mellitus in Shanghai, China
Source: J Diabetes. 2022 Dec 16;15(1):27–35. doi: 10.1111/1753-0407.13343 (PMC9870741; doi:10.1111/1753-0407.13343)

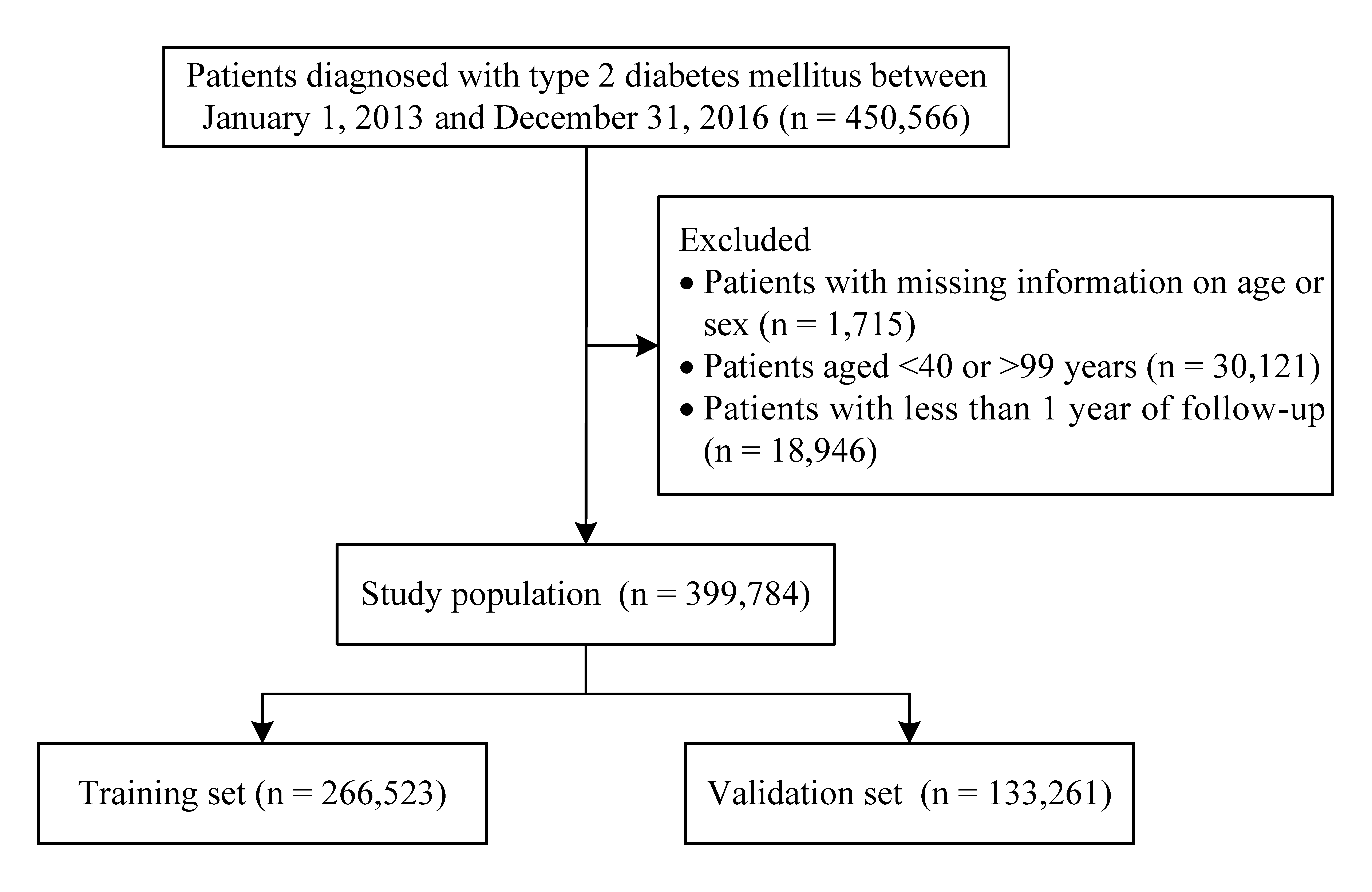

Supplement: Supplementary file 1 — Figure S1. Flowchart showing the selection process of the study population [file JDB-15-27-s001.tif]

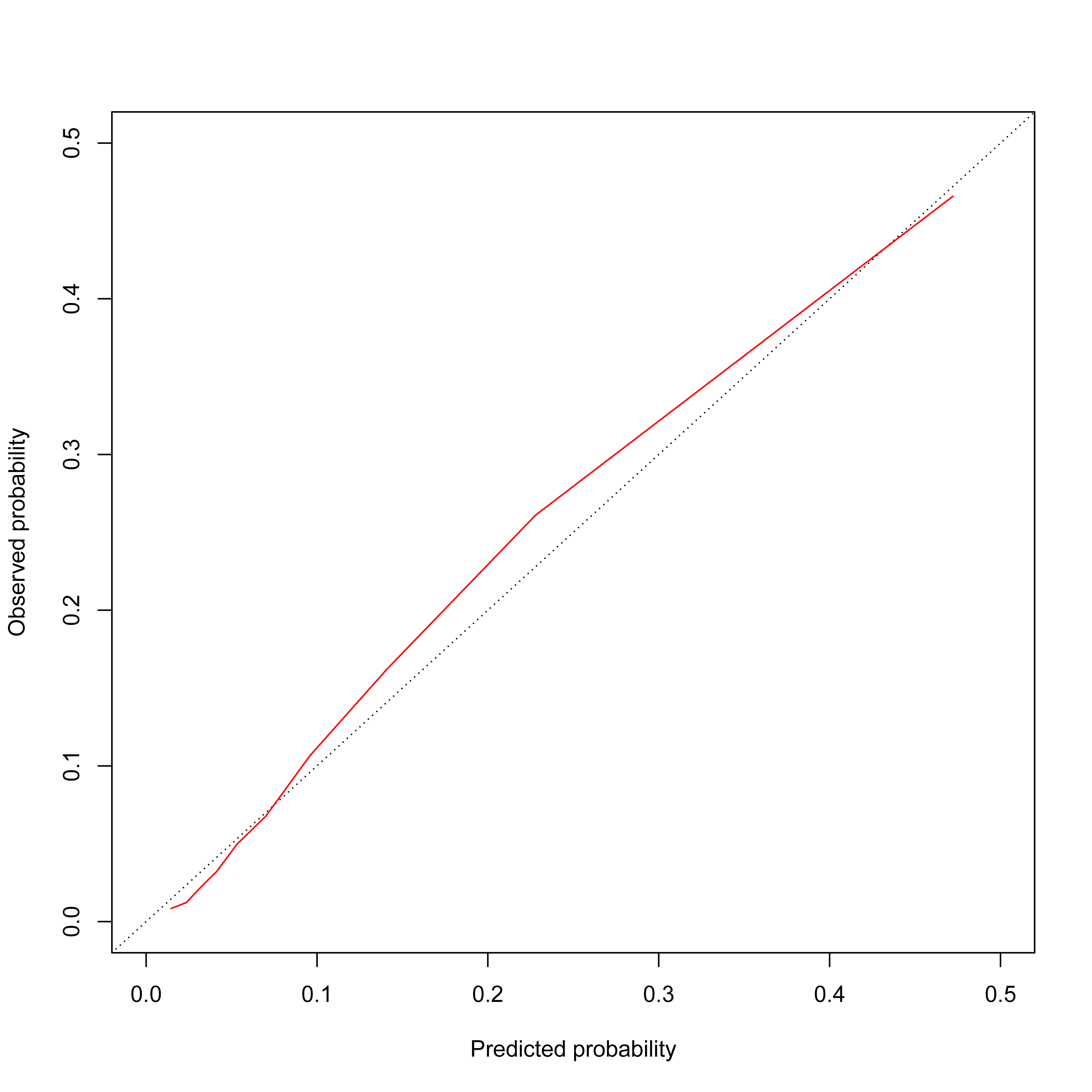

Supplement: Supplementary file 2 — Figure S2. Calibration curve of the 5‐year all‐cause mortality risk prediction model in the complete dataset [file JDB-15-27-s003.tif]
