# Supplementary material for: Developing a prediction model for all‐cause mortality risk among patients with type 2 diabetes mellitus in Shanghai, China
Source: J Diabetes. 2022 Dec 16;15(1):27–35. doi: 10.1111/1753-0407.13343 (PMC9870741; doi:10.1111/1753-0407.13343)
Supplement: Supplementary file 3 — Table S1. ICD‐10 codes for diseases Table S2. Data distribution of biochemical indicators in the original and imputed datasets [file JDB-15-27-s002.docx]

Table S1. ICD-10 codes for diseases

| Diseases | ICD-10 codes |
| --- | --- |
| Type 2 diabetes mellitus | E11.x |
| Hypertension | I10.x-I15.x |
| Dyslipidemia | E78.x |
| Diabetic complications | E11.2-E11.5, E11.7 |
| Ischemic heart disease | I20.x-I25.x |
| Peripheral vascular disease | I70.x, I71.x, I73.1, I73.8, I73.9, I77.1, I79.0, I79.2, K55.1, K55.8, K55.9, Z95.8, Z95.9 |
| Heart failure | I09.9, I11.0, I13.0, I13.2, I25.5, I42.0, I42.5-I42.9, I43.x, I50.x, P29.0 |
| Cerebrovascular diseases | G45.x, G46.x, H34.0, I60.x-I69.x |
| Dementia | F00.x-F03.x, F05.1, G30.x, G31.1 |
| Chronic lung disease | I27.8, I27.9, J40.x-J47.x, J60.x-J67.x, J68.4, J70.1, J70.3 |
| Moderate or severe kidney disease | I12.0, I13.1, N18.3- N18.5, N19.x, Z49.0-Z49.2, Z94.0, Z99.2 |
| Mild liver disease | B18.x, K70.0-K70.3, K70.9, K71.3-K71.5, K71.7, K73.x, K74.x, K76.0, K76.2-K76.4, K76.8, K76.9, Z94.4 |
| Moderate or severe liver disease | I85.0, I85.9, I86.4, I98.2, K70.4, K71.1, K72.1, K72.9, K76.5, K76.6, K76.7 |
| Cancer | C00.x-C96.x |

Abbreviation: ICD-10, *International Classification of Diseases, 10th Revision*.

Table S2. Data distribution of biochemical indicators in the original and imputed datasets

| Variables | Missing values  n (%) |  | Original dataset | | |  | Imputed datasets (range)^a^ | | |
| --- | --- | --- | --- | --- | --- | --- | --- | --- | --- |
|  |  |  | Median | IQR | |  | Median | IQR | |
|  |  |  |  | 25% | 75% |  |  | 25% | 75% |
| HbA1c (%) | 203,238 (50.84) |  | 7.10 | 6.30 | 8.50 |  | 7.10-7.10 | 6.30-6.30 | 8.40-8.40 |
| Total cholesterol (mmol/L) | 209,522 (52.41) |  | 4.63 | 3.87 | 5.42 |  | 4.65-4.66 | 3.89-3.89 | 5.45-5.46 |
| HDL-C (mmol/L) | 215,639 (53.94) |  | 1.12 | 0.93 | 1.36 |  | 1.14-1.14 | 0.94-0.95 | 1.38-1.38 |
| LDL-C (mmol/L) | 215,359 (53.87) |  | 2.78 | 2.15 | 3.43 |  | 2.80-2.81 | 2.17-2.18 | 3.46-3.47 |
| Triglyceride (mmol/L) | 211,640 (52.94) |  | 1.44 | 1.02 | 2.09 |  | 1.42-1.43 | 1.01-1.01 | 2.05-2.06 |

Abbreviations: HbA1c, glycosylated hemoglobin; HDL-C, high-density lipoprotein cholesterol; IQR, interquartile range; LDL-C, low-density lipoprotein cholesterol.

^a^ Range of the values of the 10 imputed datasets.
